# Inflammatory Bowel Disease Therapy: Beyond the Immunome

**DOI:** 10.3389/fimmu.2022.864762

**Published:** 2022-05-09

**Authors:** Claudio Fiocchi, Dimitrios Iliopoulos

**Affiliations:** ^1^ Department of Inflammation & Immunity, Lerner Research Institute Cleveland, Cleveland, OH, United States; ^2^ Department of Gastroenterology, Hepatology & Nutrition, Digestive Disease and Surgery Institute, Cleveland Clinic, Cleveland, OH, United States; ^3^ Athos Therapeutics Inc., Los Angeles, CA, United States

**Keywords:** inflammatory bowel disease, IBD, immunome, complexity, integration, systems biology, network medicine

## Introduction

### Definition of Immunome

Inflammation is the most common biological occurrence in any living organism’s life, and it can be both beneficial and harmful. Under homeostatic conditions inflammation is finely orchestrated, controlled and necessary for defense and return to normality whereas, under pathological conditions, inflammation is excessive, uncontrolled and causes tissue, organ or system damage. An inflammatory response can be caused by loss of structure, loss of regulation or loss of function (1) but, regardless of its cause, is always a highly convoluted process. This had led to notion of “immunome”, the sum of all cellular and molecular structures arbitrating inflammation. The term immunome was proposed in 1999 by Pederson, who defined it as “*a limit concept, viz the totality of rearranged antibody and antigen receptor genes present in all living humans*.” (2). With continuous progress in many fields of biology and technology this definition has evolved and has acquired an expanded all-inclusive connotation.

### Current View of the Immunome

We know now that the immunome is far more than antibodies and antigen receptors, and is now variably defined as a composite of leukocyte immunophenotypes (3), the cellular environment mediating immune responses (4), a comprehensive single cell immune profiling (5), and the future of blood testing (6). The immunome is much more than an assembly of immune cells, their functions, products and effects, but includes any immune and non-immune component participating in a response, the interactions among all these components and the environment where the interactions take place. This all-inclusive definition has fundamental implications because it expands and diversifies how immune responses are initiated, executed, regulated, and terminated or prolonged. When applied to inflammation, this broad definition renders any inflammatory disease remarkably complex, not only regarding the mechanisms but also the interventions to modify its outcome. The complexity of the immunome, or in fact of any other ome, can be tackled with advanced computational tools, and many exist that allow disease pattern identification, patient subtyping, network construction and visualization, controllers discovery, therapeutics linkages and drug toxicity prediction (7).

### The Immunome in Autoimmune and Chronic Inflammatory Diseases

The immunome is the central arbitrator of all chronic inflammatory diseases (immune-mediated inflammatory diseases - IMIDs), where myriads of factors come together to trigger inflammation and sustain it for extended periods or even a lifetime (8). The pathogenesis of IMIDs is a perfect example of immunome complexity. Rather than a molecularly defined entity, each IMID is a heterogeneous syndrome (9), where heterogeneity is shaped by the respective immunomes containing both unique and shared cellular and molecular mechanisms (10). There are various IMIDs where the investigation of the immunome has helped to better understand the underlying pathophysiology, like in rheumatoid arthritis (11), systemic lupus erythematosus (12), type 1 diabetes (13), and psoriasis (14), just to mention a few. The practical value of these studies is illustrated by a recent study of rheumatoid arthritis, where the prospective analysis of immune cells found in a simple fingerstick coupled with clinical follow-up allowed the detection of circulating blood cells that precede a clinical flareup (15). This indicates that the analysis of immunome components detectable in a blood sample, like cells, proteins, metabolites, etc., may reveal key diagnostic and prognostic information in multiple IMIDs.

### The IBD Immunome

In inflammatory bowel disease (IBD) the immunome is the key effector of phlogosis, but it is also useful for diagnosis, patient stratification and disease staging. To achieve this the IBD immunome must be integrated with other omes such as the exposome, genome, epigenome, transcriptome, proteome, metabolome, and microbiome (16). All these omes are interdependent, and all need to be taken into account collectively to uncover IBD pathogenesis (17). Therefore, the IBD immunome cannot be analyzed in isolation as it is currently done with sophisticated but primarily descriptive single cell technologies and spatial transcriptomics (18–22). These methodologies provide information on cell phenotypes, receptors, transcripts and tissue localization as well as associations with degree of inflammation or IBD subtype. However, results are inconsistent and of limited value because they are described in a biological vacuum that excludes other omes. This generates dissimilar IBD immunome “signatures”, further complicating its interpretation and pathogenic role. Despite these drawbacks, the IBD immunome is still considered the best target for therapeutic interventions because both Crohn’s disease (CD) and ulcerative colitis (UC) are IMIDs mediated by dysregulated immunity. This perception explains the development of a growing assortment of medications aimed at blocking specific single components of the IBD immunome (23), and this narrow unidirectional approach has improved clinical outcomes. However, beneficial outcomes are restricted to <50% of patients, are unpredictable and accompanied by significant side effects, and are lost with time (23). Most notably, the response rates are similar regardless of which component of the immune response is being affected (24), demonstrating that targeting single components of the IBD immunome is an unsatisfactory form of therapy. This raises fundamental questions: 1) *Why is the current therapeutic approach to IBD inadequate?* and 2) *How can the therapeutic approach to IBD be improved?* This opinion article will try to address both questions.

## Why Is the Current Therapeutic Approach to IBD Inadequate?

By targeting the same components of the immunome in all IBD patients implicitly assumes that that the gut inflammatory process is the same in all patients. This could not be further from the truth. Humans display an enormous immune diversity driven by genes, age, sex, cohabitation, environment and season, and this diversity evolves over time (25, 26). Immunity is not set but a continuum of archetypes with innate and adaptive immune plasticity that incessantly adapts to fluctuating needs (27). Consequently, the composition and function of the immunome undergoes incessant changes even under short periods of time (28, 29). This variation is not unique to the immune system, but an intrinsic property of all biological systems that explains heterogeneity of mechanisms and variability of responses (30). Thus, one must remember that the IBD immunome also continuously adjusts, and what mediates IBD at one stage may not at another stage, and therapeutic interventions must adjust and target different components at different times. The number of factors that modify the IBD immunome is enormous, and only a few examples will be mentioned.

### Exposome

The word *exposome* is used to define the endless diversity of exposures that humans undergo during life. Such exposures include *exogenou*s chemicals, foods, psychological and physical stressors and all the *endogenous* factors resulting from the subsequent biological responses (31). That environmental factors affect IBD is known, as shown by smoking and diet (32–34), but the list of environmental factors conditioning or modulating IBD keeps growing, including air pollutants, e-cigarettes, proton pump inhibitors and many others (35–38). A link between the exposome and the IBD immunome is the aryl hydrocarbon receptor, a transcription factor receptive to a variety of endogenous and exogeneous ligands (39, 40), and able to modulate intestinal immunity (41).

### Microbiome

The link between the gut microbiome and IBD is firmly established by plentiful experimental and clinical evidence (42, 43), creating a key connection with the immunome (44). This can happen directly through the response of immune cells to microbial antigens and metabolites but also indirectly through the action of antibiotics (45), non-antibiotic drugs (46), genes (47) and pollutants (48). Each of these agents alters the microbiome and the associated immune response in unique ways, modifying the IBD immunome and creating the need to customize therapies to its modification.

### Proteome and Metabolome

A less investigated but no less important connection exists between the immunome and metabolism, and immunometabolism in particular. Metabolism is at the core of all biological functions, and this is also true for the immune response, since metabolism regulates immune cell quiescence, proliferation and differentiation (49, 50). This is relevant to the global function of the immunome in health and disease (51), and several experimental approaches to interrogate immunometabolism are already available, such as computational metabolic and genomic metabolic models, that allow to investigate exposome metabolic dependencies of immune cells, disease regulators, and multiomics integration (52). Reports on metabolism and immunometabolism in IBD are relatively few, but several metabolic perturbations have been detected in the stools, serum, plasma and mucosal tissues of CD and UC patients, all of them potentially impacting on the IBD immunome (53).

### Epigenome

The investigation of the IBD genome has been extensive (54), but less so for the IBD epigenome. Epigenetic modifications occur throughout lifetime (55) and are critically important because they reprogram the function of immune cells (56), and in so doing the function of the immune system under physiological and pathological conditions (57). Multiple mechanisms induce epigenetic changes, such as those induced by microRNAs, methylation status, and the posttranscriptional “epitranscriptomic” mRNA modification (58–61). The epigenetic modulation of the IBD immunome is just starting to be explored, but it’s crucial importance will become evident when viewed under the magnifying glass of the *biological integration* discussed in the following paragraph.

### Biological Integration

The above unidirectional interplays are only one aspect of the actual biological interfaces going on in IBD. In fact, while one particular ome modulates the immunome, it also impacts on all others omes which, in turn, also affect the immunome. The result is an intricate and reciprocally communicating interactome (62), as shown by numerous examples: a whole host of environmental insults induces a host-microbiome adaptation which alters gene expression and immunity (40, 63, 64); lifestyle, antibiotics, common drugs and environmental components modify the gut microbiome (46, 48, 65), which consecutively modifies the immunome; diet impacts on microbiome-immune interactions (66), and gene-microbiota interactions occur in IBD (47); genetic and environmental factors interact with age and sex in shaping the methylome (67), and DNA methylation mediates genetic risk of IBD (58). Considering the almost infinite number of these biological correlations, any intervention that targets a single component of the IBD immunome is bound to fail to significantly alter its composition or normalize its function.

## How Can the Therapeutic Approach to IBD be Improved? Go Beyond the Immunome

The advent of anti-TNF biologics unquestionably represented a major step forward in IBD management, followed by the development of multiple other biologics and small molecules targeting other cytokines, integrins, receptors, signaling and homing molecules, alone or in combination (68). What all these therapies have in common is the blockade of a specific but single component of the IBD immunome, an approach that reaches a <50% therapeutic success (24). To overcome this plateau in drug efficacy combination therapies are being proposed (69), but they are unlikely to substantially improve efficacy and may induce more negative side effects (70). This relentless search for new IBD drugs using the present single target philosophy (71) resembles what has been done in Alzheimer’s disease, where amyloid has been the only therapeutic target for years. Many agents have been developed to neutralize amyloid, and the scientific and pharmaceutical communities insist in pursuing this avenue even though no concrete evidence of benefit has ever been demonstrated (72). In face of the complexity of the IBD immunome and its constant modifications due to the influence of other omes, one must conclude that it is time to go beyond the immunome to develop breakthrough therapeutic strategies (23, 73).

### Unbiased Integration-Based New IBD Target Discovery

Disease heterogeneity and a disproportionate emphasis on single immune targets are at the base of the low response rate of current IBD therapies, and it is time to go beyond the immunome to develop precision medicine drugs by combining molecular and clinical data. In oncology, the NIH TCGA consortia have outlined the roadmap to precision medicine and how to identify novel drug/gene targets in patient subgroups. For example, integration of genomic, epigenomic, transcriptomic and clinical information from colon cancer patients identified a particular molecular subtype with high microsatellite instability (74). This subtype responds to immunotherapy with a significant longer progression-free survival (75), validating the multi-omic molecular integration approach for target discovery. A similar roadmap should be applied to IBD, aiming first at identifying IBD molecular subgroups. By doing this, these IBD patient subgroups can benefit from custom therapy that specifically targets the underlying molecular mechanisms unlike the current non-specific anti-inflammatory or immunosuppressive drugs or single target biologic agents. Admittedly, integration of molecular and clinical data is challenging because of a variety of factors such as biological complexity, multifactorial etiopathogenesis, patient heterogeneity, clinical and temporal variability, and biosample collection and size (7). Nevertheless, efforts in this direction are currently under way, like the discovery of a new drug that specifically targets the hub of an interactome present in a particular subgroup of UC patients (unpublished data). Additional efforts in this direction are being made (76–78), but they need longitudinally collected biomaterials from well characterized patients. It is also essential to develop novel computational tools tailored to multi-omic IBD datasets (43, 79). The ultimate aim of these efforts will result in the identification of gene, protein, metabolite or microbe hubs that will become new IBD drug targets and/or companion diagnostics.

## Conclusion

**Figure 1:**
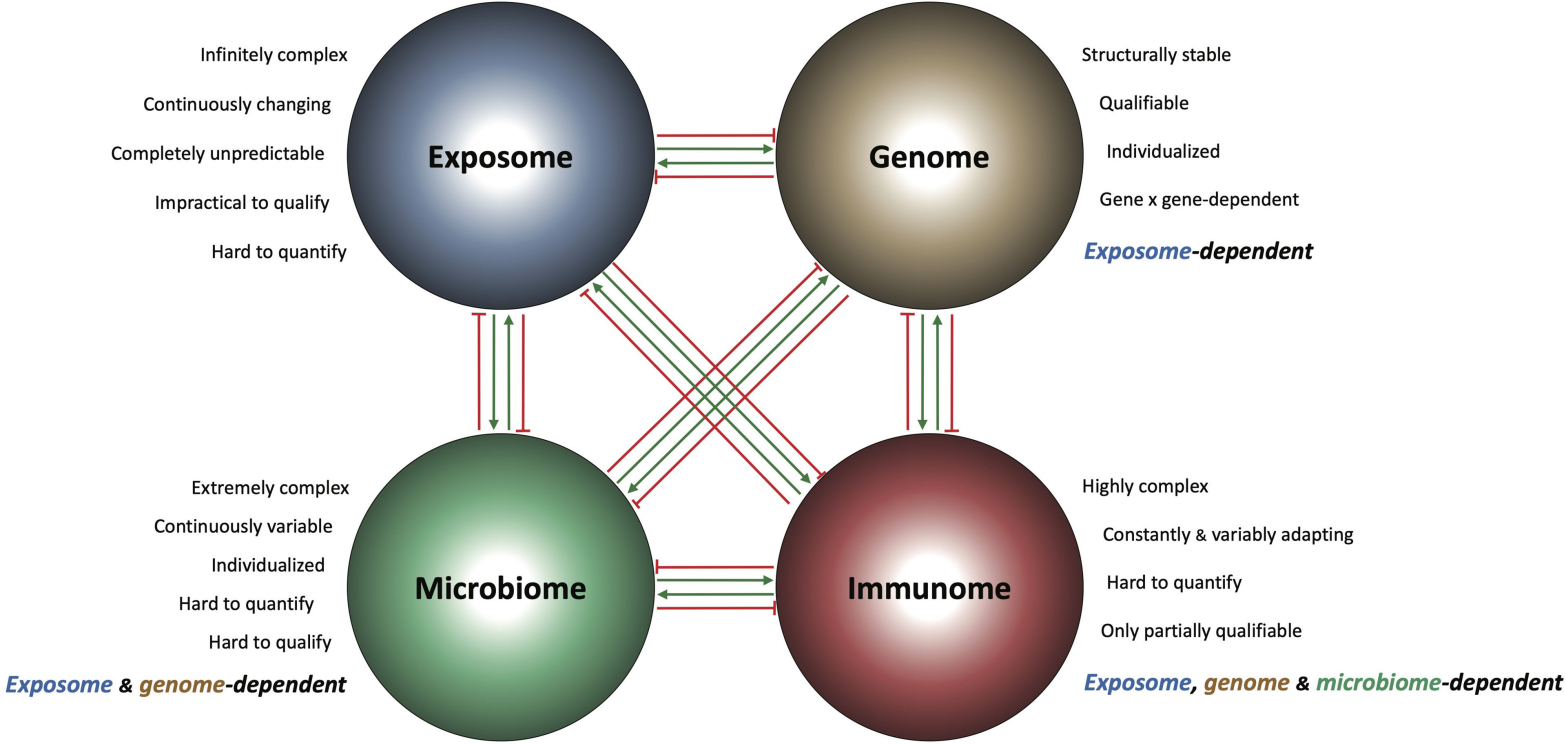
Schematic representation of the IBD interactome and its components. Each of the four major pathogenic IBD omes (exposome, genome, microbiome and immunome) is intrinsically complex and influences the composition and behavior of the other omes in a stimulatory (green arrows) and/or inhibitory (red arrows) way. This creates a biological interconnectedness and interdependence among the IBD omes, where the function of the genome is exposome-dependent, the function of the microbiome is exposome and genome-dependent, and the function of the immunome is exposome, genome and microbiome-dependent.

Current therapeutic strategies for IBD are largely focused on blocking a single component of the IBD immunome at one time, and results are considered positive when the clinical response is statistically greater than that of placebo. When primary endpoints are not reached then the results are deemed “promising” because some patients showed an indication of response, and larger studies are advocated. In adopting this “thought collective” attitude the IBD community and pharmaceutical industry prolong the present *status quo* and set aside the reality that IBD is a life-long, continuously evolving, heterogeneous and extremely complex disease. Not only is the IBD immunome extremely complex, but so are all other interconnecting omes, creating the convoluted IBD interactome responsible for chronic gut inflammation. **Figure 1** lists the factors responsible for the high complexity of each ome and illustrates the boundless interactions among them. Complex problems require complex solutions (80), and these demand integrate the IBD immunome with all other relevant pathogenic omes to find the disease controlling hub(s).

## Author Contributions

CF and DI contributed equally to all components of this opinion article and agree to be accountable for its content. All authors read and approved the final manuscript.

## Conflict of Interest

Author DI was employed by Athos Therapeutics Inc.

The remaining authors declare that the research was conducted in the absence of any commercial or financial relationships that could be construed as a potential conflict of interest.

## Publisher’s Note

All claims expressed in this article are solely those of the authors and do not necessarily represent those of their affiliated organizations, or those of the publisher, the editors and the reviewers. Any product that may be evaluated in this article, or claim that may be made by its manufacturer, is not guaranteed or endorsed by the publisher.
